# Perceptions of Endocrine Therapy in African-American Breast Cancer Survivors: Mixed Methods Study

**DOI:** 10.2196/23884

**Published:** 2021-06-11

**Authors:** Sara Donevant, Sue P Heiney, Cassandra Wineglass, Benjamin Schooley, Akanksha Singh, Jingxi Sheng

**Affiliations:** 1 College of Nursing University of South Carolina Columbia, SC United States; 2 Department of Integrated Information Technology College of Engineering and Computing University of South Carolina Columbia, SC United States

**Keywords:** mHealth, breast cancer survivors, medication adherence, cultural considerations, mobile health applications

## Abstract

**Background:**

Although the incidence of breast cancer is lower in African-American women than in White women, African-American women have a decreased survival rate. The difference in survival rate may stem from poor endocrine therapy adherence, which increases breast cancer recurrence. Therefore, accessible and culturally sensitive interventions to increase endocrine therapy adherence are necessary.

**Objective:**

The purpose of this concurrent convergent mixed methods study was to provide further data to guide the development of the proposed culturally sensitive mHealth app, STORY+ for African-American women with breast cancer.

**Methods:**

We recruited 20 African-American women diagnosed with estrogen-positive breast cancer and currently prescribed endocrine therapy. We used a concurrent convergent data collection method to (1) assess the use of smartphones and computers related to health care and (2) identify foundational aspects to support endocrine therapy adherence for incorporation in a mobile health app.

**Results:**

Overwhelmingly, the participants preferred using smartphones to using computers for health care. Communicating with health care providers and pharmacies was the most frequent health care use of smartphones, followed by exercise tracking, and accessing the patient portal. We identified 4 aspects of adherence to endocrine therapy and smartphone use for incorporation in app development. The factors that emerged from the integrated qualitative and quantitative data were (1) willingness to use, (2) side effects, (3) social connection, and (4) beliefs about endocrine therapy.

**Conclusions:**

Further research is needed to develop a culturally sensitive app for African-American women with breast cancer to improve adherence to endocrine therapy. Our work strongly suggests that this population would use the app to connect with other African-American breast cancer survivors and manage endocrine therapy.

## Introduction

Breast cancer is the second-most common cancer among women in the United States and results in the second-highest cancer death rates [1]. Earlier detection through screening and advances in treatments have contributed to an increase in the 5- and 10-year breast cancer survival rates [2]; however, African-American women have decreased survival rates and higher mortality rates compared to White women despite lower incidence rates [3]. One potential explanation for the differences in survival rates and mortality among women with estrogen-positive breast cancer is adherence to long-term endocrine therapy, which may last for 5 to 10 years [4]. Endocrine therapy, which blocks estrogen receptors in breast cancer cells, can reduce recurrence by 40% and lower the risk of dying by one-third [5]. Overall, African-American women have low long-term adherence rates to endocrine therapy [6,7]. Therefore, interventions to assist this vulnerable population with endocrine therapy adherence are essential [4,8].

We conducted a literature review on interventions that improve endocrine therapy adherence, which identified 2 consequential gaps in existing intervention research: use of education only interventions and a lack of cultural adaptations [9-16]. Next, we examined commercially available cancer and medication adherence mobile health (mHealth) apps as a possible option to address these gaps and assist African-American women with endocrine therapy adherence [17-21]. Unfortunately, available cancer and medication adherence apps also have several areas that cause concern: (1) a lack of adequate development or testing in clinical practice [17,22,23], (2) a lack of input from patients or providers resulting in significant usability problems [24-26], and (3) a lack of scientifically valid information, with the majority of cancer-related apps created to promote a pharmacy or organization and not to assist African-American women with endocrine therapy adherence [20,26]. Importantly, over 90% of medication adherence mHealth apps were simple reminders and not effective in improving adherence [20]. Cancer mHealth apps research has explored functionality and acceptability, but only one evaluated effectiveness or clinical outcomes [17-19].

To address these gaps in endocrine therapy adherence and mHealth apps for medication adherence, we propose to extend our earlier teleconferencing work, Sisters Tell Others and Revive Yourself (STORY), a culturally tailored intervention that connected African-American women with breast cancer to support and educated them during initial diagnosis and treatment [27]. We wanted to explore how STORY components could assist endocrine therapy adherence in a more accessible platform such as an mHealth app. The purpose of this concurrent convergent mixed methods study [28] was to provide further data to guide the development of the proposed culturally sensitive mHealth app, STORY+ for African-American women with breast cancer. Our initial work has been reported [14,29-34]. The aims of this study were to (1) assess the use of smartphones and computers related to health care in African-American women with breast cancer, and (2) identify foundational aspects to support endocrine therapy adherence for incorporation in an mHealth app.

## Methods

### Overview

The concurrent convergent mixed methods design of our study incorporated the simultaneous collection, analysis, and interpretation of qualitative and quantitative data to inform the design of an mHealth app to increase adherence to endocrine therapy in African-American women with breast cancer. We recruited 20 African-American women with a diagnosis of estrogen-positive breast cancer and prescribed endocrine therapy. We conducted an ex-ante study (ie, before the design and construction of the app). Prior to the start of the study, the University of South Carolina Institutional Review Board approved the study (Pro00085557).

### Sampling Strategy and Inclusion Criteria

We combined 2 sampling schemes (ie, criterion and convenience) to select our sample. These strategies followed the works of Onwuegbuzie and Collins [35], who established typologies for sampling designs in social science research and suggested appropriate sample sizes. We also used design science research criteria (ie, innovation and evolution) to establish the legitimacy of our sample size. A sample of 20 or fewer for formative research is often employed [36,37]. We needed women who met our enrollment criteria, but we wanted heterogeneity in the sample. We used a random number generator to select potential participants for recruitment from a pool of 1577 patients. We reviewed the potential participants’ medical records and selected the first 50 patients who met the inclusion criteria. This process created our first batch of potential participants. From previous recruitment efforts, we had estimated that 50 potential participants would supply our recruitment goal of 20 participants. We planned to recruit until we reached informational redundancy [38,39] (ie, both qualitative and quantitative data provided no new information). If the initial batch did not supply sufficient participants, the process was to be repeated, and data collection would be continued.

Inclusion criteria were African-American women who were 18 years of age or older, had been diagnosed with estrogen-positive breast cancer in the past 10 years, were currently prescribed endocrine therapy, and were able to speak and understand English. Reading English was not a requirement because the researcher read the informed consent and questions to the participants. Exclusion criteria were individuals with a diagnosis of psychosis, with significant cognitive impairment, or undergoing current treatment for another cancer excluding squamous cell (any type).

### Recruitment and Retention

**Figure 1:**
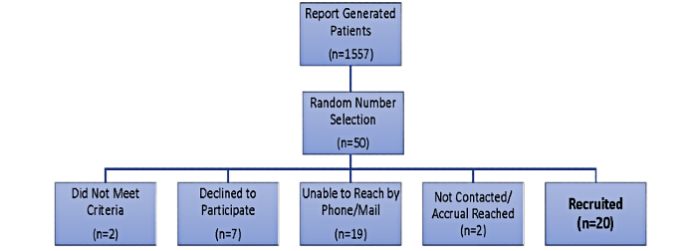
Study flow diagram.

Women who were eligible based on the inclusion and exclusion criteria were recruited through a comprehensive oncology outpatient practice in South Carolina (500 new breast cancer patients annually). Our recruitment plan was covered by a Health Information Portability and Accountability Act waiver with the oncology outpatient cancer physician practice. We used the Heiney-Adams Recruitment Framework to guide our recruitment efforts [40]. The principal investigator mailed a personal letter to potential participants with a colorful and readable frequently asked questions flyer using the STORY logo (designed by focus group participants in STORY). Within 5 days of the mailing, a racially concordant researcher contacted participants by telephone. The researcher followed a script that asked if the patient had a diagnosis of estrogen-positive breast cancer and had been prescribed tamoxifen or an aromatase inhibitor. To improve recruitment and retention in the study, participants received a gift card for their time and effort. We successfully recruited 20 patients. From the original list of 50 potential participants, 19 could not be reached by phone or mail, 7 declined to participate, 2 did not meet the criteria, and 2 were not contacted because accrual was reached (Figure 1).

### Data Collection

The research team, which included nurses and software computer engineers, developed a data collection tool using Research Electronic Data Capture (REDCap [41]; Vanderbilt University). The data collection tool consisted of a qualitative interview guide (Multimedia Appendix 1) and quantitative (open- and closed-ended) questions (Multimedia Appendix 2). Data collection, which we digitally recorded, began with structured questions and ended with semistructured questions. The researcher pilot tested the REDCap data collection tool with 1 African-American breast cancer survivor before the data collection process, resulting in minor adjustments of the tool based upon the feedback. This pilot testing established the ease of data collection using REDCap during an interview, not reliability or validity.

Once the researcher received verbal consent from the participant during the telephone conversations, she scheduled the assessment appointment at the participant’s choice of location, usually in the home or a meeting room in the local library. During this assessment appointment, the researcher (1) read all interview questions to the participant, (2) recorded the responses directly in the REDCap data collection tool via a tablet for the quantitative questions, and (3) digitally recorded the qualitative interview questions and participants’ responses. A professional transcriptionist transcribed the deidentified recordings verbatim.

### Data Analysis

#### Quantitative Data

We exported the data from REDCap into Excel (Microsoft Inc) for analysis. For continuous variables, we calculated means, standard deviations, and ranges. For categorical variables, we calculated frequencies and percentages.

#### Qualitative Data

We used thematic analysis [42]. No software was used to analyze the qualitative data. Briefly, data analysis began by listening to the digital recordings of participant responses to the semistructured interview questions. Next, 2 researchers read and reread the transcripts and began open line by line coding of the data. We coded significant and salient phrases and words within each transcript. We identified and discussed themes from these codes until consensus was reached.

#### Data Integration

For clarity, we organized the findings by aim. Most findings are reported with quantitative data and supporting qualitative quotes [39]. In some instances, qualitative themes emerged that were not explored in the quantitative questions and vice versa. In these cases, the results are displayed separately.

## Results

### Sample Description

Participants’ (n=20; age: mean 59 years, SD 10) mean length of time diagnosed with breast cancer was 4 years (Table 1). Most participants had stage 1A breast cancer (9/20, 45%). Over 50% of participants (11/20) were prescribed tamoxifen, which did not change during their treatment period. The length of time on endocrine therapy was evenly split between more than 2 years (10/20, 50%) and 2 years or less (10/20, 50%).

**Table 1:** Participant characteristics.

Characteristics			Value, n (%)
**Age (years)**			
	Mean (SD)	59 (10)	
	Range	46-82	
**Length of time since diagnosis/recurrence^a^ (years)**			
	Mean (SD)	4 (4)	
	Range	1-17	
**Stage of breast cancer**			
	0	4 (20)	
	1A	9 (45)	
	1B	2 (10)	
	2A	1 (5)	
	2B	2 (10)	
	3A	1 (5)	
	4	1 (5)	
**Medications**			
	Tamoxifen	11 (55)	
	Anastrozole	8 (40)	
	Letrazole	1 (5)	
**Length of time on medication**			
	<2 years	10 (50)	
	>2 years	10 (50)	
**Always prescribed same medication**			
	Yes	14 (70)	
	No	6 (30)	

^a^One patient experienced recurrence within the past 5 years.

### Smartphone and Computer Use for Health Care

Overwhelmingly, the participants preferred using a smartphone for health care over using a computer.

I pretty much do everything on my cell phone because it’s always with me.

Participant 13

With respect to health care use, participants reported communicating with health care providers and pharmacies most frequently, followed by exercise tracking, and accessing the patient portal (Table 2).

**Table 2:** Smartphone and computer use for health care.

Use	Quantitative data			Qualitative data
	Smartphone, n (%)	Computer, n (%)	Quotations	
Communicate with health care provider	15 (75)	2 (10)	“... sometimes my provider will send me a message or tell me they put something out on the portal for me to go and check.” [Participant 14]	
Communicate with pharmacy	13 (65)	0 (0)	”My pharmacist will contact me via text mail.”[Participant 7] “The prompt I got today from [the pharmacy] was that I needed a refill and would I allow them to call my doctor. Of course which I said yes.” [Participant 12]	
Exercise tracking, coaching, or management	7 (35)	0 (0)	“The only health aid I use is a Fitbit to help keep up with my steps. I’m supposed to make at least 10,000 steps per day.” [Participant 13] “I got an Engage app from my job with my insurance and stuff. It is a program we have on the job. Well, I went through the program with a dieting thing. It was to help you lose weight and it sets you up. They send you a scale. You weigh every day. They give you a coach. She logs in every day with different ideas and stuff for you to do meals to plan and it also helps, but my insurance will come down instead of paying a surcharge.” [Participant 18]	
Patient portal	7 (35)	7 (35)	“I use my patient portal...” [Participant 1] ”But I do a lot on my phone. If I want to check my medical records, I go to the patient portal for the various organizations that have my medical records where I can check.” [Participant 19]	
Connect or manage wearables	5 (25)	0 (0)	“I’m just mainly [monitor] with my Fitbit.” [Participant 5] “I use the Fitbit...” [Participant 19]	
Medication management or reminders	2 (10)	0 (0)	“Just the pharmacy’s [app] for my medication reminders.” [Participant 13] “I feel if I ever took that reminder off my phone that I will miss one [medication dose]...”[Participant 4]	
Diet tracking, coaching, or management	1 (5)	3 (15)	“...Samsung app for fitness. You can put your food diet, your food thing in it. It counts your calories, your steps, your pulse, and all of that. And it’s the app that automatically comes with a Samsung phone.”[Participant 1] “Well, I went through the program with a dieting thing. It was to help you lose weight and it sets you up. They send you a scale. You weigh every day. They give you a coach. She logs in every day with different ideas and stuff for you to do meals to plan and it also helps, but my insurance will come down instead of paying a surcharge.” [Participant 20]	
Personal health records	1 (5)	2 (10)	—^a^	

^a^A representative quotation is not available.

### Factors

#### Overview

We identified 4 aspects of endocrine therapy adherence and smartphone use that may guide app development. The factors that emerged from the integrated qualitative and quantitative data included (1) willingness to use, (2) side effects, (3) social connection, and (4) beliefs about endocrine therapy.

#### Willingness to Use an App for African-American Breast Cancer Survivors

The majority of participants (17/20, 85%) stated that they would use an mHealth app to assist with endocrine therapy adherence. They specifically mentioned an online community of other breast cancer survivors and African-American–tailored graphics (ie, emojis, videos). The qualitative interviews provided additional details on the importance of an app for African-American breast cancer survivors:

On the [majority] of apps, I see it is still [for] white [women]. I don’t normally see too many blacks that I can reach out to. I would love to share with an African-American female [with breast cancer] what I’ve been through. Every app that I [see] dealing with breast cancer shows white [women], and I would love to talk to African-American women, women of color.

Participant 9

I think the other thing is, when people see people like them, not like they’re not out there, because they are, then they’d be more apt to reach out.

Participant 19

It seems like a lot of women that have it [breast cancer] are ashamed to tell other women about it [breast cancer] and you’re in the dark when you get it [breast cancer]. The only time you find out about it [breast cancer] is when you’re in a conversation and you say, oh, I had breast cancer, then she’ll share hers [diagnosis] with you. But other than that, they [women] don’t like to say this, open with their answer. No, and so to see that and then see what other women are going through it’ll help me too.

Participant 8

I feel like with my story...Sharing it [my story] with others... it [sharing my story] might brighten their day or it might help them to understand that just because you had cancer it’s nothing to be ashamed of.

Participant 10

#### Self-Reported Side Effects

In patients’ self-reported experiences (ie, reports were not verified by health care providers) with the side effects of endocrine therapy (Table 3), almost one-third (6/20, 30%) of participants stated that they called their health care provider about the side effects, with 15% (3/20) reporting they made an appointment with the health care provider to discuss the side effects. In addition, 80% (16/20) of participants talked with a family member, friend, or significant other about the side effects.

**Table 3:** Self-reported endocrine therapy side effects.

Side effect	Quantitative data, n (%)	Quotations
Hot flashes	17 (85)	“So, sometimes I put a cool towel around my neck, but it’s just, I just get hot around the neck. It’s like I’m on fire.” [Participant 13] “I’ve been using a lot of air-conditioning. But it [hot flash] doesn’t last long, and I usually get it like once a day. At any time. And it will last about like 15 minutes and then it’s gone.” [Participant 6]
Bone/joint pain	13 (65)	“I actually had to get referrals for some of the pain. I actually had to end up going to the orthopedic. I had problems with my left hand. The pain, I couldn’t hold anything [in my left hand] and I was in severe pain.” [Participant 5] “So, then you look up like, all right, God, my joints are hurting, what the hell?” [Participant 12]
Fatigue or lack of energy	11 (55)	“The biggest problem I had with side effects, the fatigue I would try to kind of pace myself when I’m doing certain things especially after coming home from work instead of just getting right into it doing some laundry or doing, I kind of take a break.” [Participant 14]
Weight gain	11 (55)	“I have gained much weight. I hate it [weight gain].” [Participant 17] “I can deal with the weight gain because I can continue to walk and it just helps me see the nature outside,...” [Participant 4]
Hair thinning	10 (50)	“...I did experience, um, hair thinning.” [Participant 14]
Increased sweating	10 (50)	—^a^
Mood swings	10 (50)	—
Leg cramps	9 (45)	—
Dry skin and/or eyes	7 (35)	—
Insomnia	7 (35)	“...some nights I sleep five hours and some, but I know if I got at least six to seven hours that was a good night’s sleep for me because there are times when I’m say, but if I’m up at night...” [Participant 14]
Loss of sex drive	7 (35)	“God, I gotta have an orgasm, this is ridiculous. This is pissing me off. That almost made me quit.” [Participant 12] “I do have to say that I was gonna talk to my doctor about, you know, anything she can do with the part that comes to like my loss of sex drive, like, that bothers me a lot because I know, like, I know my husband, not that anything is wrong.” [Participant 4]
Constipation	6 (30)	—
Depression	5 (25)	“When I started, the depression almost made me quit. For sure. Because I’m not a depressed person.” [Participant 12]
Vaginal dryness	5 (25)	—
Vision problems	5 (25)	—
Back pain	4 (20)	“Like I said, when I get up in the morning my back hurts me, but I go to a 9:30 class at Rec Center, so by the time I’ve exercised it’s not hurting me as much. I was leaving there [Rec Center] and going two times per week for the dry needling and stuff and that has helped...” [Participant 15]
Dizziness	4 (20)	“It, um, you do get a little dizzy with them because taking this medicine. I mean, I think any hormone medicine will make you feel a little woozy at times. Um, it’s not, it’s not every day though. I don’t have it every day. I have it every now and then.” [Participant 8]

^a^A representative quotation is not available.

#### Beliefs About the Value of Endocrine Therapy

There were many motivators, especially external motivators, for patients to continue endocrine therapy. The most prominent motivators for endocrine therapy adherence included the desire to live longer (16/20); children (16/20); and religion, church, or a higher being (15/20). Other motivators included friends (13/20), extended family (13/20), and significant others (11/20). Both quantitative and qualitative data demonstrated similar themes in motivators to endocrine therapy adherence, including increased mortality and living for family.

I must say life. Because if I didn’t take it [endocrine therapy] then that would make my body more [likely] to [have] what I’ve had in the past. So, when it was told for me to take it this is what I needed to do and that is what I’m doing. So, I’m gonna say life.

Participant 9

I take it [endocrine therapy] because they say I need to take it with the medicine because of my situation with the breast cancer. They say I have to take that. I didn’t want to take it because I said I felt like it wasn’t helping me. And she [the doctor] said, well, yeah you need to take that. You have to continue taking that. So, I’m just saying like they say I gotta take it [endocrine therapy]...Because it’s gonna help me with my situation. I’m gonna do what I gotta do for me.

Participant 3

Well, I was told that it [endocrine therapy] would keep the cancer away. It’s a tool, I guess it’s considered as a chemo agent to keep your hormonal level down. That’s what was explained to me.

Participant 13

Well, I know I have to take it [endocrine therapy] for the next five years. That’s part of my treatment. I know it’s part of my treatment, so I’m willing to do whatever is necessary to follow the treatment plan.

Participant 6

My family. My grandson. My son. I only have one child and he’s everything to me and my grandson - he gives me life. My grandson gave me life. I got diagnosed the year that he was born, so it was like, oh no, no, no, no, no, I gots to be here for him.

Participant 4

#### Social Connections

Qualitative and quantitative data supported the importance of connecting with other African-American women with breast cancer. Participants valued digital social connections and face-to-face interactions. They used smartphones to connect and interact with other individuals including survivors with 85% (17/20) using smartphones for communication (ie, phone calls, email, texting). Participants used smartphones for instant messaging (12/20, 60%) and video chat (11/20, 55%). Facebook was the most popular social media platform among the participants, with 65% (13/20) accessing Facebook via smartphones and computers. Instagram was the next popular social media platform with 25% (5/20) accessing Instagram via smartphone and 15% (3/20) accessing Instagram via computer.

Interviews with the participants substantiated the variety of ways African-American women with breast cancer connected and their importance in the breast cancer survivor journey. Participants discussed the need for connecting with other African-American breast cancer survivors and described supportive interactions.

We talk a lot because actually in my job it was quite a few of us that were diagnosed in a short period of time.

Participant 1

We have a group, we still hang together after 52, 53 years, two of my friends, my classmates had already had breast cancer, so I certainly reached out to them to talk to them, and that helped, so that was it.

Participant 5

Well, I only have a few people I connect with and we all work in the same department. We [are] all in it with the same type of cancer.

Participant 13

I’d like to see what other women, African-American women are going through and see if, you know, if we’re all going through the same thing or if it’s, you know, different.

Participant 2

It’s [Livestrong Program] through the Y[MCA] and that’s a program that you should share with other African-American women. I was the only African-American woman that was in it.

Participant 15

But, when you talk to people, you really want support not sympathy. A person can empathize with you... But not sympathy and you need someone that’s going to be strong in the support when you are talking to them.

Participant 5

Find their support system whether it be family, group meetings and there’s a bunch of them out there... Church, co-workers. I had all of that. Definitely, don’t try to go through it alone. Have your support system. That’s the most important thing because there’s gonna be days you just feel like, why me. I mean, there’s just gonna be those days. That’s all I can say. You need somebody to talk to, talk to them.

Participant 17

## Discussion

### General

This concurrent convergent mixed methods study provides additional data to guide the development of STORY+. We assessed the use of smartphones and computers related to health care in the targeted population and identified foundational mHealth features to support endocrine therapy adherence. Overall, the participants used smartphones more frequently than they used computers for health care, especially for social interactions such as social media, messaging, and email. This finding suggests that African-American breast cancer survivors are more likely to use an mHealth app than they are to use a webpage via computer to assist with endocrine therapy adherence. Overwhelmingly, African-American breast cancer survivors use smartphones in managing their health through communication with their health care provider and pharmacy to promote their health.

While fewer participants reported using mHealth apps for medication tracking and health care record management, qualitative results indicated that combining social and cultural features with mHealth functions would be desirable. Other researchers have also identified the need for racial and cultural content [43-49]. Our study verifies that African-American women do not have a culturally sensitive mechanism to track symptoms and discuss symptoms with health care providers. We concluded that foundational STORY+ features should include the following: an online community of other African-American breast cancer survivors, tailored graphics, information about prescribed endocrine therapy and its potential side effects, and a method for tracking side effects, sharing the frequency and severity of side effects, and recording medication adherence. Previous findings [50] have also supported the use of interactive features to promote positive health outcomes—participants overwhelmingly supported the development of an mHealth app to assist with endocrine therapy adherence and social connections with other African-American breast cancer survivors.

### Limitations

The study is not generalizable beyond the immediate needs of the research team; however, the results of this study provide a foundation for the mHealth app development, STORY+. The literature suggests that other breast cancer survivors experience similar endocrine therapy side effects [51].

### Conclusions

This concurrent convergent mixed methods study established the use of smartphones by African-American breast cancer survivors for health care management. We also identified foundational features for STORY+. This work recognizes that one app does not fit all needs and advances the science of cultural and racial appropriate mHealth apps. Future work will include the development and testing of STORY+ for African-American breast cancer survivors to promote endocrine therapy adherence.

## Appendix

Multimedia Appendix 1 Qualitative questions.

Multimedia Appendix 2 Quantitative survey.

## Abbreviations

mHealth: mobile health
REDCap: Research Electronic Data Capture
STORY: Sisters Tell Others and Revive Yourself
