# Supplementary material for: Perceptions of Endocrine Therapy in African-American Breast Cancer Survivors: Mixed Methods Study
Source: JMIR Form Res. 2021 Jun 11;5(6):e23884. doi: 10.2196/23884 (PMC8235283; doi:10.2196/23884)
Supplement: Multimedia Appendix 1 [file formative_v5i6e23884_app1.docx]

# Supplementary File 1: Qualitative Questions

1. What tools help you remember to take your endocrine therapy medicine?
   1. If you take other medications, it is ok to share what you do to help you take any of them.
2. What techniques or prompts help you to remember to refill your prescription?
3. **If participant selected performing health care functions or tasks on cell phone:** You stated that you use your cell phone for health care. What health related apps do you use?
4. **If participant selected performing health care functions or tasks on computer:** You stated that you use your computer for health care. What health related programs do you use/access?
5. Have you ever considered using a computer or cell phone to assist with medication management?
   1. **If yes to computer:** How has medication management on your cell phone worked for you?
   2. **If yes to cell phone:** How has medication management on your cell phone worked for you?
6. What is the most important reason to take your endocrine therapy?
7. How do you connect with other African-American women with breast cancer?
8. How did you handle the side effects of endocrine therapy?
9. Is there any other information I should have asked about endocrine therapy that I did not ask?
10. If you had access to a free medication app that focused on endocrine therapy medication for African-American women with breast cancer (African-American emojis, videos with other African-American breast cancer survivors, survivors, online community of other African-American cancer survivors), would you be interested in using the app?
    1. Why would you be interested? Or Why would you not be interested?
