# Supplementary material for: Perceptions of Endocrine Therapy in African-American Breast Cancer Survivors: Mixed Methods Study
Source: JMIR Form Res. 2021 Jun 11;5(6):e23884. doi: 10.2196/23884 (PMC8235283; doi:10.2196/23884)
Supplement: Multimedia Appendix 2 [file formative_v5i6e23884_app2.docx]

# Supplementary File 2: Quantitative Questions

## Questions on Endocrine Therapy Medication

1. What is the name of the endocrine therapy medicine (anti-estrogen ) you are currently taking?
2. Anastrozole (Arimidex)
3. Exemestane (Aromasin)
4. Fulvestrant (Faslodex)
5. Goserelin (Zoladex®)
6. Megace
7. Letrozole (Femara)
8. Leuprolide (Lupron®)
9. Raloxifene
10. Tamoxifen
11. Toremifene (Fareston)
12. Decline to answer
13. Other (provide name)
14. Unsure
15. How long have you been taking endocrine therapy?
16. < 6 months
17. 6 months to 1 year
18. 1 year to 2 years
19. 2 years to 3 years
20. 3 years to 4 years
21. 4 years to 5 years
22. 5 years to 6 years
23. 6 years to 7 years
24. 7 years to 8 years
25. 8 years to 9 years
26. 9 years to 10 years
27. >10 years
28. Decline to answer
29. Unsure
30. Have you always taken this drug or did your doctor prescribe a different one?
31. Always taken this drug
32. Provider prescribed a different drug
33. Decline to answer
34. Unsure
35. If provider prescribed a different drug, what endocrine therapy medication did you take before?
36. Anastrozole (Arimidex)
37. Exemestane (Aromasin)
38. Fulvestrant (Faslodex)
39. Goserelin (Zoladex®)
40. Megace
41. Letrozole (Femara)
42. Leuprolide (Lupron®)
43. Raloxifene
44. Tamoxifen
45. Toremifene (Fareston)
46. Decline to answer
47. Other (provide name)
48. Unsure
49. What (if any) side effects have you experienced from your medication (select all that apply)?
50. Back pain
51. Blood clots
52. Bone/joint pain
53. Bone thinning or osteoporosis
54. Constipation
55. Depression
56. Diarrhea
57. Dizziness
58. Drowsiness
59. Dry skin and eyes
60. Endometrial cancer
61. Fatigue or lack of energy
62. Flu-like symptoms
63. Headaches
64. Hot flashes
65. Increased sweating
66. Injection site pain
67. Insomnia
68. Leg cramps
69. Loss of sex drive
70. Low calcium levels
71. Mood swings
72. Nausea/vomiting
73. Rash
74. Sore throat
75. Stomach/abdominal pain
76. Stroke
77. Swelling
78. Vaginal discharge/bleeding
79. Vaginal dryness
80. Vaginal irritation
81. Vision problems
82. Weight gain
83. Do not experience side effects
84. Decline to answer
85. Other (please specify)
86. Were the side effects so bad that you needed to call your provider or have an appointment?
87. Yes
88. No
89. Decline to answer
90. Not applicable
91. Did you talk to a family member, spouse/significant other, or friend about the side effects?
92. Yes
93. No
94. Decline to answer
95. Not applicable
96. What or who motivates/encourages or influences you to take your hormonal therapy (select all that apply)?
97. Child
98. Community
99. Extended family (i.e., cousin, aunt/uncle, niece/nephew, grandparent, etc.)
100. Friend(s)
101. Living longer
102. Parent
103. Pets
104. Other breast cancer patient(s)
105. Sibling
106. Spouse or significant other
107. Religion, church, higher being
108. Other (please specify)

## Questions on Technology Use

1. Do you have cell phone (select all that apply)?
2. Yes, personal Apple cell phone
3. Yes, personal Android cell phone
4. Yes, work Apple cell phone
5. Yes, work Android cell phone
6. Yes, other personal cell phone
7. Yes, other work cell phone
8. No, I do not own a cell phone
9. Decline to answer
10. Please check all factors that influence your selection of a cell phone (select all that apply)?
11. Brand
12. Cost
13. Ease of use
14. Number of features (hardware or software)
15. Physical size or weight
16. Decline to answer
17. Not applicable
18. How often do you use cell phone?
19. Use cell phone daily
20. Use cell phone several times a week (but not daily)
21. Use cell phone several times a monthly (but not weekly)
22. Rarely/less than monthly/only for emergencies
23. Decline to answer
24. Not applicable
25. What functions or actions do you commonly perform on the cell phone (select all that apply?
26. Communication (e-mail, calls, texting, etc.)
27. Daily planning (schedule, tasks, appointments)
28. Entertainment (movies, TV, reading books, music, games)
29. Financial transaction (banking, shopping, etc.)
30. GPS, navigation, maps, directions
31. Health care (diet, patient portal, exercise, wearables, health monitoring device)
32. Office applications (word processing, spreadsheets, etc.)
33. Searching Internet
34. Shopping
35. Social media (Facebook, Twitter, etc.)
36. Taking pictures or videos
37. Decline to answer
38. Not applicable
39. Other (please describe)
40. **If communication selected**: Please select all the types of communications you do on your cell phone (select all that apply)?
41. Chats
42. E-mail
43. Facetime, other video chat or video conference
44. Instant messaging
45. Phone calls
46. Text messaging
47. Decline to answer
48. Not applicable
49. **If social media selected**: Please select all the types of social media you use on your cell phone (select all that apply)?
50. Facebook
51. Instagram
52. LinkedIn
53. Snapchat
54. Twitter
55. WhatsApp
56. Decline to answer
57. Not applicable
58. Other (please describe)
59. **If entertainment selected**: Please select all the types of entertainment you use on your cell phone (select all that apply)?
60. Audiobooks
61. Digital books
62. Games
63. Movies
64. Music
65. Read books
66. TV
67. Decline to answer
68. Not applicable
69. Other (please describe)
70. **If financial transactions selected**: Please select all the types of financial transactions you use on your cell phone (select all that apply)?
71. Banking
72. Calculator
73. Pay bills
74. Shopping
75. Transfer money
76. Decline to answer
77. Not applicable
78. Other (please describe)
79. **If office applications selected**: Please select all the types of office applications you use on your cell phone (select all that apply)?
80. Journaling
81. Notes
82. Spreadsheets
83. Word processing
84. Presentations
85. Decline to answer
86. Not applicable
87. Other (please describe)
88. **If health care selected**: Please select all the cell phone technologies you use for your health care (select all that apply)?
89. Communicate with pharmacy
90. Communicate with health care provider
91. Connect or manage wearables (FitBit, Apple Watch, etc.)
92. Diet tracking, coaching, and/or management
93. Disease management or symptom tracking
94. Exercise tracking, coaching, and/or management
95. Home monitoring devices (BP, glucose, etc.)
96. Medication management/reminders
97. Patient portal(s)
98. Personal health records
99. Decline to answer
100. Not applicable
101. Other (please describe)
102. Do you have computer (select all that apply)?
103. Yes, personal computer
104. Yes, work computer
105. No, I do not have access to a computer
106. Decline to answer
107. Please check all factors that influence your selection of a computer (select all that apply)?
108. Brand
109. Cost
110. Ease of use
111. Number of features (hardware or software
112. Physical size or weight
113. Decline to answer
114. Not applicable
115. Other (please describe)
116. How often do you use a computer?
117. Use computer daily
118. Use computer several times a week (but not daily)
119. Use computer several times a month (but not weekly)
120. Rarely/less than monthly/only for emergencies
121. Decline to answer
122. Not applicable
123. What functions or actions do you commonly perform on the computer (select all that apply?
124. Communication (e-mail, calls, texting, etc.)
125. Daily planning (schedule, tasks, appointments)
126. Entertainment (movies, TV, reading books, music, games)
127. Financial transaction (banking, shopping, etc.)
128. GPS, navigation, maps, directions
129. Health care (diet, patient portal, exercise, wearables, health monitoring device)
130. Office applications (word processing, spreadsheets, etc.)
131. Searching Internet
132. Shopping
133. Social media (Facebook, Twitter, etc.)
134. Taking pictures or videos
135. Decline to answer
136. Not applicable
137. Other (please describe)
138. **If communication selected**: Please select all the types of communications you do on your cell phone (select all that apply)?
139. Chats
140. E-mail
141. Facetime, other video chat or video conference
142. Instant messaging
143. Phone calls
144. Text messaging
145. Decline to answer
146. Not applicable
147. **If social media selected**: Please select all the types of social media you use on your cell phone (select all that apply)?
148. Facebook
149. Instagram
150. LinkedIn
151. Snapchat
152. Twitter
153. WhatsApp
154. Decline to answer
155. Not applicable
156. Other (please describe)
157. **If entertainment selected**: Please select all the types of entertainment you use on your cell phone (select all that apply)?
158. Audiobooks
159. Digital books
160. Games
161. Movies
162. Music
163. Read books
164. TV
165. Decline to answer
166. Not applicable
167. Other (please describe)
168. **If financial transactions selected**: Please select all the types of financial transactions you use on your cell phone (select all that apply)?
169. Banking
170. Calculator
171. Pay bills
172. Shopping
173. Transfer money
174. Decline to answer
175. Not applicable
176. Other (please describe)
177. **If office applications selected**: Please select all the types of office applications you use on your cell phone (select all that apply)?
178. Journaling
179. Notes
180. Spreadsheets
181. Word processing
182. Presentations
183. Decline to answer
184. Not applicable
185. Other (please describe)
186. **If health care selected**: Please select all the cell phone technologies you use for your health care (select all that apply)?
187. Communicate with pharmacy
188. Communicate with health care provider
189. Connect or manage home monitoring devices (BP, glucose, etc.)
190. Connect or manage wearables (FitBit, Apple Watch, etc.)
191. Diet tracking, coaching, and/or management
192. Disease management or symptom tracking
193. Medication management/reminders
194. Patient portal(s)
195. Personal health records
196. Decline to answer
197. Not applicable
198. Other (please describe)
199. Please select all the features you like on the programs/apps that you use (select all that apply)?
200. Ability to communicate with others within the app (app community)
201. Bar code or QR scanning
202. Connect with other devices (headphones, wearables, scales, car, etc.)
203. Connect to social media within an app
204. Customizable colors and themes (look and feel)
205. Customization or personalization (alerts, reminders, feedback)
206. Easy registration/set up process
207. Easy to use format
208. Educational information within the app (text, videos)
209. Features and functions work properly and consistently
210. Graphs and tables about data/information in the app
211. Intuitive icons and buttons
212. Readable test
213. Search within the app
214. Secure and password protect log-on
215. Shares information between apps or other systems (cloud, electronic medical record, etc.)
216. Understandable text/information
217. Decline to answer
218. Not applicable
219. Other (please describe)
